# A family-based education program for obesity: a three-year study

**DOI:** 10.1186/1471-2431-7-33

**Published:** 2007-10-22

**Authors:** Rita Tanas, Renzo Marcolongo, Stefania Pedretti, Giuseppe Gilli

**Affiliations:** 1 Pediatric Department, Azienda Ospedaliera-Universitaria S. Anna, Ferrara, Italy; 2 Department of Clinical and Experimental Medicine, Azienda Ospedaliera-Università, Padova, Italy; 3 Therapeutic Education Laboratory, Padova, Italy; 4 Programmer, Health Physics Department, Azienda Ospedaliera-Universitaria S. Anna, Ferrara, Italy

## Abstract

**Background:**

The epidemic of obesity is increasing in all countries. However, the number of controlled studies focusing on childhood obesity, with a long follow-up is still limited. Even though Behavioral Therapy shows some efficacy, it requires a prolonged teamwork that is not always available in public health settings. In addition, Behavioral Therapy is not always accepted. We describe a new intensive and sustainable family-based, Therapeutic Education program for childhood obesity.

**Methods:**

Controlled clinical study: a family-based Therapeutic Education program without dietetic prescription involving overweight and obese children/adolescents, without evident psychological troubles, and their families. The program consisted of three clinical and therapeutic education sessions, carried out by a single physician. Further sessions were carried out every six months in the first year and then every year.

Study population: 190 overweight children, 85 treated with a therapeutic education program (45 males and 40 females, mean age of 10.43 ± 3) with an average BMI% of 154.72 ± 19.6% and 105 matched children, treated with traditional dietary approach.

Children's Body Mass Index (BMI) % and BMI Standard Deviation Score measured at baseline and after a three year-follow-up, were compared. Statistical tests: ANOVA-RM (repeated measures) controlled for distribution by Kolmogorov-Smirnov, Bartlett's test or correspondent non-parametric procedures, X^2 ^tests or Fisher's exact test and simple linear regression.

**Results:**

After a follow-up of 2.7 ± 1.1 years, 72.9% of the children who followed the Therapeutic Education Program obtained a BMI% reduction, compared to 42.8% of children who followed the traditional dietary treatment. Weight reduction was good in moderately obese children and in the severely obese. In addition, a smaller proportion of children treated with therapeutic education had negative results (BMI increase of >10%) compared to those treated with dietary approach (11.8% vs. 25.7%); finally, periodic phone calls reduced the drop-out rate in the therapeutic education group.

**Conclusion:**

These results indicate the efficacy and sustainability of the Therapeutic Education program, that was completely carried out by a single pediatrician; in addition, it met with an elevated participant acceptance, suggesting a convenient therapeutic solution for skilled pediatricians and selected obese children, when Behavioral Therapy is not available or teamwork is poor.

## Background

In the past twenty years, the prevalence of pediatric obesity has risen significantly in Italy [1], as it has worldwide, causing alarm in both medical and political circles. To date, proposed therapeutic approaches for obesity have met with little success. In fact, according to a recent Cochrane Library review [2], standard effective therapy for obesity is not yet available. Many authors affirm that current treatments remain largely unsuccessful [3]. Simple dietetic interventions have a high percentage of failure [4], with initial weight loss followed by frustrating regain or overweight persistence and increase. Even though behavioral therapy seems to provide some results, the few clinical studies with long follow-up in adults show that, within 2–3 years, they usually miss the target of weight reduction [5].

When considering children, some authors report successful results following family-based multi-professional behavioral treatment programs [6-8]. Although these are considered the gold-standard therapy, they require a number of professional resources and a prolonged teamwork [9,10] that are not always available in public health facilities nor easily accepted by families.

Medical literature reports only a small number of pediatric studies with a long-term follow-up [6-8,11]. Even if they are successful in producing weight loss, most of the treated children fail to became non obese; in addition, they usually report a high drop-out rate: Braet and Golley report 20% [8,12] and Savoye found 24% [11]. Finally, all authors notice that the drop-out rate may be even greater when considering children with more severe obesity; moreover it tends to increase during treatment, and it seems inversely related to treatment efficacy [13].

Follow-up studies suggest that, as in adults, also in obese children and adolescents more realistic weight goals are to be pursued. Nowadays, adult obesity is considered a chronic disease, which, although not cured, can be managed through intensive, lifelong treatment programs. In this view, a steady weight loss, ranging from 5 to 10% [14,15] and lasting for at least two years, is associated with significant health benefits and is generally considered a good therapeutic result.

Unfortunately, when considering children/adolescent obesity, despite the Israel Consensus Statement [16] in 2005 which proposed that any z-BMI (Body Mass Index z-score) decrease should be evaluated as a good therapeutic result, universally accepted therapeutic goals are still lacking.

In recent years, some authors have been supporting the idea that, compared to weight fluctuation or increase, the mere maintenance of a steady weight should also be regarded as a good result [8]. Moreover, it also seems that slight weight reductions might be realized avoiding dietary restraint that in adolescence is often associated with the onset of eating disorders [17].

Moreover, therapeutic programs that have been developed in academic settings, by committed and motivated professionals, are quite difficult to implement in ordinary healthcare facilities, where human resources are usually limited, overburdened and poorly motivated [18].

In the attempt to overcome, at least in part, some of the above mentioned problems, we devised an "intensive" Therapeutic Education Program (TEP), including only essential learning content and involving the active engagement of both families and their children, without clinically evident psychiatric troubles [19]. The program was conceived based on the authors' long-standing experience in the treatment of weight-related diseases and on their conviction about the importance of teaching a healthy, yet pleasant lifestyle, and of treating weight-related teasing [11,20,21].

Medical literature reports only few studies on the education treatment of overweight children with a long follow up and without the prescription of a diet. Braet and coll. by a behavioral and education treatment obtained a BMI (Body Mass Index) % decrease of 11% for 4.6 years. In particular, they report that also children who received one-session of advice obtained a significant benefit (BMI% -6.2%) [8].

We report the results of a clinical study involving 190 consecutive children, referred by primary care physicians to our Endocrinology Unit over a three-year period; 85 children received a short therapeutic group education, without any diet prescription, while the remaining 105 received diet prescription.

## Methods

### Participants

Our therapeutic education program was started seven years ago at the Pediatric Department of Ferrara Hospital. Eligible children included only Caucasian overweight and obese children and adolescents, aged 3 to 18 years, with a follow up of at least one year. Children with secondary obesity and psychiatric problems were excluded. An informed oral consent was obtained from children's parents/caregivers before enrolment. The study was approved by the Ethics Committee of Ferrara.

From March 2000 to March 2003, 85 families of obese or overweight children (45 males and 40 females, 47 in pre-pubertal and 38 in pubertal stage), joined the therapeutic education program (TEP), attending it for at least 1 year (range 1–5.6 years, mean 2.7 ± 1.1); only 2 children dropped-out before 1 year, due to serious family problems. The age of children ranged from 3.1 to 18.3 years (mean 10.43 ± 3), their mean BMI% was 154.72 ± 19.6%, their mean BMI Standard Deviation Score (BMI SDS) was 2.54 ± 0.8. Before starting the program, 7 out of 85 children were overweight, 78 obese, 52 of them severely obese. Their parents' BMI was 27.56 ± 3.6 (mothers 26.61 ± 4.6, fathers 28.5 ± 4.2).

In the same period, 105 families of overweight or obese children (47 males and 58 female, aged 4.5–15.5 years, mean 10.19 ± 2.8) followed a traditional dietetic program (DT). Their mean BMI% was 141.78 ± 19.4; their mean BMI SDS was 2.10 ± 0.7. Before starting the DT, 24 children were overweight and 81 obese, 35 of them severely obese. Their parents' BMI was 27.51 ± 3.7. On average, the starting BMI% value of the study group was higher compared to that of control-group (mean 154.7 vs 141.8%, p < 0.05). The children of the two groups were matched for age, gender, parents' BMI and follow-up time. The study population was entirely Caucasian, of random socio-economic extraction and with average education and average knowledge of diet and nutrition.

This is a retrospective, non-randomized, non case-controlled clinical study. Case allocation between the two groups was completely fortuitous; indeed, when families called our center asking for advice and consultation, they were allowed to make a completely free and uncoerced choice between different healthcare professionals who work in two separate clinics in the same unit and to whom the public has access in a random fashion based on availability.

### Procedure

#### Measures

Body height, expressed in 0.1 cm intervals, was measured by a Harpenden's stadiometer, while body weight, expressed in 0.1 kg intervals, was determined by a medical balance beam scale. Height and weight were used to calculate BMI: kg/m^2^.

Percentage overweight (BMI%) was calculated by the following formula: BMI/BMI at the 50^th ^BMI percentile ×100.

BMI expressed as a standard deviation score (BMI-SDS), was calculated according to Luciano's Italian references [22], by usual least mean square method [23].

In most cases heights and weights were assessed in our laboratory; self-reported values, as assessed by family pediatricians were also accepted [24] when children and families were unable or, due to different reasons, could not attend assessment sessions during follow up.

Overweight was defined within values of BMI ranging from the 85^th ^to the 94^th ^percentile, and obesity for a value of BMI ≥ 95^th ^percentile. Obese children with a BMI ≥ 99^th ^percentile were considered "severely obese [25]."

Participants' BMI, BMI%, and BMI-SDS were recorded at baseline and after the follow-up period in order to assess their evolution and to make a comparison between their values [26].

Stages of puberty were assessed according to Tanner [27], from stage 1 (puberty not started) to stage 5 (ended puberty).

#### Therapeutic Education Program (TEP)

We named our program *"The Balloon Game *[19]" recalling the action of reshaping a toy balloon in a longer rather than larger way. We made it with the aim of exchanging the feelings of shame and guilt usually linked to obesity, for those of fun and blamelessness of an amusing game. In agreement with the reported evidence [28-31], our TEP includes three steps, carried out by a single skilled pediatrician:

• *Initial assessment and education session *(single family group). During this first session, taking about one hour, the pediatrician assesses children's eating behavior, physical activity, psychological condition (including self-esteem and body-image), knowledge and beliefs about obesity and its treatment. The physician also explores the point of view, the eating behavior and the life-style of children's family members (parents or caregivers). Then the pediatrician fosters their motivation to enter the therapeutic program, encouraging them to change their eating behavior, physical activity pattern and home environment, placing the focus on well-being and on a healthier and more pleasant way of living rather than on weight loss. Even if not obese, all family members are warmly invited to improve their eating style and physical attitude.

• *Therapeutic education session for parents/caregivers in small groups*. During this session, taking about two hours, after carrying out brainstorming about the concept of "obesity", the pediatrician discusses participants' knowledge and beliefs about health, food, physical activity and lifestyle. Then, with the aid of simple explanations and illustrations, gathered in a self-help booklet, the physician teaches the group about the importance of parents and friends as models for the children's behavior. She then teaches them some basic self-monitoring skills and how to correctly self-assess daily food and caloric intake, according to different situations and individual needs. Additionally, basic positive reinforcement techniques (that do not include food or money) are proposed and families are taught how to adjust their lifestyle and environment (e.g., how to reduce access to high-fat and low-nutrient dense foods, how to shop and cook healthier foods, how to increase access to physical activity while avoiding behaviors that prompt inactivity, such as TV watching, playing computer games, etc.), just as in behavioral therapy. At the end, all participants fill up a questionnaire, investigating their learning, opinions and feelings about the session.

• *Second assessment session (single family group)*. After 2 months, the pediatrician meets again with each child and their parents/caregivers for about 40 minutes, to provide them with information about the obesity-related risk of the child. The physician also gives them positive feedback for every behavioral or BMI% improvement. She then assesses their knowledge and self-management skills and explores barriers and problems encountered during the first phase of the program. She finally proposes to each child and family group a personalized follow-up schedule that includes:

- A clinical assessment every six months for children with a low risk of obesity-related complications (physical, psychological or social), without a family history of obesity and related complications, with normal laboratory data and satisfactory clinical results (improved lifestyle and/or BMI% decrease).

- A clinical assessment every four months for teenagers and severely obese children or when initial satisfactory results are lacking and the risks of obesity-related complications are high.

- An education session every two weeks, during the first three months, for children who show body-image dissatisfaction or "emotional eating" at the initial clinical-education assessment.

With the exception of children seeking medical advice for personal problems, after the first year, the follow-up schedule includes a clinical assessment every year. Drop-out families receive a phone call, without questionnaires, every year to reinforce their motivation of resuming follow-up program and to record their self-reported anthropometrical measures (as assessed by their family pediatrician) and lifestyle changes.

During clinical follow-up, the pediatrician explores every possible increase in physical activity and the adoption of a healthier eating behavior by the children and the family group (i.e., the assessment of some key points such as the reduction of sedentary behavior, the increase in sport or physical activity, the changes in eating behavior, etc.). Finally, with the help of a short questionnaire, she asks their advice to improve the TEP.

After a three year follow up, the pediatrician assesses the children's quality of life with a questionnaire about their emotional, social and academic functioning; she also explores both their difficulties and achievements. Finally, she openly probes the children for the temptation to leave the program.

#### Dietetic Therapy (DT)

It includes a complete clinical assessment, an advice on physical activity and a professional dietetic consultation with diet prescription; the follow up schedule is the same as in the study group, but without telephone follow-up.

#### Evaluation criteria

Body weight and fat may vary according to the influence that genetic, psychological, social and environmental factors exert on children and their families [32].

The study was aimed at the assessment of the efficacy of the TEP in the reduction or stabilization of BMI% and BMI SDS, over a long-term follow-up period. It was also intended to make a comparison between TEP and DT.

Then, in consideration of the natural history of pediatric obesity and of the current absence of universally acknowledged and shared goals for its treatment, we scored clinical outcome as follows:

##### Positive or Successful

when no BMI% increase was observed (Normal-weight: BMI <85th percentile at follow-up).

##### Stabilized

when a very small BMI% increase was observed (0.01–4.99% from baseline).

##### Negative

when a BMI% increase was observed (≥ 10% from baseline).

Weight variations were expressed as a difference between mean BMI% and BMI-SDS at baseline and at the last follow-up.

#### Statistical analysis

Results were analyzed using SPSS v.8.0. (SPSS Inc, Chicago IL, USA), and Statgraphics v.4.0 (STSS, Inc. Rockville, MD, USA) and with ANOVA-RM, as the basic procedure for data analysis, once the method feasibility was verified by distribution tests and Levene's test of variance equality. TEP and DT plus gender and obesity-degree, all together or separately, represented the "between factors" while the "before" and "after" treatment times always played the role of the "within factor".

X^2 ^tests were also employed to verify the possible occurrence of differences in the number of cases becoming positive, negative or normalized following the two treatments.

We also employed linear regression to find weight variation trends related to some parameters, such as age, puberty stage, follow-up-span. An α-error of 5% was set as study significance threshold.

## Results

**Figure 1:**
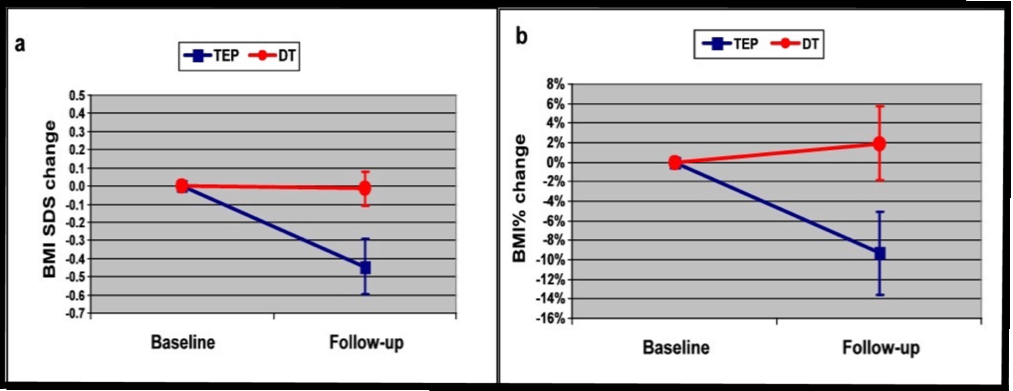
**BMI SDS (a) and BMI% (b) changes from baseline to follow-up in the two therapeutic groups**. TEP: Therapeutic Education Program: Number children 85. DT: Dietetic Therapy: Number children 105. BMI%: Body Mass Index %. BMI SDS: BMI Standard Deviation Score. a: p < 0.01. b: p < 0.05.

Figure 1 and Table 1 summarize the results observed in the two groups at the end of the follow-up.

**Table 1:** Baseline and changes of anthropometric measures after 3 years, following TEP and the DT

**Measured Parameter**	**At baseline**	**After a 3 year follow-up**
Therapeutic Education Program (n.85 children)		

BMI%	154.72 (19.6)	-9.32 (15.3)*
BMI-SDS	2,54 (0.8)	-0.44(0.7)**

Dietetic Therapy (n.105 children)		

BMI%	141.78 (19.4)	+1.89 (13.3)*
BMI-SDS	2.10 (0.7)	-0.01(0.5)**

*p < 0.05

** p < 0.01

BMI%: Body Mass Index Percent;

BMI-SDS: BMI Standard Deviation Score.

Data are given as mean (SD).

Following TEP, 72.9% (62) of children had positive results, seven of them became normal-weight. In the TEP group, as a whole, the BMI% decreased by 9.32 ± 15.3 BMI SDS 0.44 ± 0.7); in particular, the decrease was 4.86 ± 9.2% (baseline 126.97 ± 5.1%) (BMI SDS decrease 0.30 ± 0.6) among *overweight *children and 9.72 ± 15.7 (baseline 157.22 ± 18.5%), (BMI SDS decrease 0.46 ± 0.7) among the *obese*. The number of obese children decreased by 16.5% (14) and that of severely obese children by 19% (16) (Table 2); 11.8% of children (10) stabilized their weight.

**Table 2:** Children distribution by obesity degree from baseline to follow-up following TEP and DT

**Obesity Degree**	**Therapeutic Education Program (TEP)**		**Dietetic Therapy (DT)**	
	**Baseline**	**Follow-up**	**Baseline**	**Follow-up**

Overweight	n. 7	n. 14 (+8.2%)	n. 24	n.19 (-4.8%)
Obesity (including severe obesity)	n. 78	n. 64 (-16.5%)	n. 81	n.78 (-2.9%)
Severe obesity*	n. 52	n. 36 (-18.8%)	n. 35	n.35 (0%)
Normal weight	n.0	n.7 (8.2%)	n.0	n.8 (7.6%)

* p < 0.01

TEP: Therapeutic Education Program

DT: Dietetic Therapy.

Negative results were observed in 11.8% of children (10) (60% females, aged 3.1–15.5 years, mean 9.34 ± 3.7); they all were obese and two of them became severely obese.

In the DT group, 42.8% (45) of children obtained positive results and eight of them became normal-weight at the end of the follow-up. All children had an average BMI% increase of 1.89 ± 13.3% (BMI SDS remained nearly unchanged; it decreased by 0.01 ± 0.5), *overweight *children's BMI% increased by 1.36 ± 9.8% (BMI SDS remained nearly unchanged, increasing by 0.04 ± 0.5) and *obese *children's BMI% increased by 2.04 ± 14.3% (BMI SDS remained nearly unchanged; it decreased by 0.03 ± 0.5). The number of obese children decreased only by 2.85% (3), while that of severely obese children remained unchanged. 22.9% (24) of children stabilized their weight.

Negative results were observed in 25.7% (27) of children (5 overweight and 22 obese, 59% females, aged 5.25–14.1 years, mean 8.98 ± 3.1) and 4 became severely obese during the follow-up.

There is a significant difference between the percentages of reduction of severely obese children following the two treatments (**X**^2 ^test p < 0.001).

The BMI% at baseline showed a positive correlation with age (linear regression p < 0.05) in the children of the two groups.

After follow-up, the BMI% of pre-pubertal children belonging to the TEP group decreased by 5.48 ± 13.9% (BMI SDS reduction of 0.42 ± 0.8), while the BMI% of pubertal children in TEP group decreased by 14.07 ± 15.8% (BMI SDS reduction of 0.48 ± 0.6); there was no statistical difference. The BMI% reduction positively correlates with the age (p < 0.05) and the stage of puberty, ranging from 1 to 5 (p < 0.01), but not with follow-up span.

The total number of "Positive" or "Successful" children (i.e., with a stable BMI%) in the TEP group was significantly higher (**X**^2 ^test p < 0.01), while that of "Negative" children (i.e., with a BMI% increase ≥ 10%) remained significantly lower in TEP compared to that of DT group (**X**^2 ^test p < 0.05). No significant difference (**X**^2 ^test) was seen in the two groups between children who normalized their weight.

The total numbers of "Positive" and "Stabilized" children was high both in TEP (72) and DT group (69), but with a significant difference (**X**^2 ^test p < 0.01), (84.7% vs 65.7%). BMI% values decreased by 13.28 ± 13% in the TEP group and by 5.18 ± 9.1% in the DT group; while BMI SDS value decreased by 0.55 ± 0.7 in the TEP group and only by 0.16 ± 0.4 in the DT group.

In particular, comparing results after TEP and DT, a significant decrease in BMI% and BMI-SDS was observed both when children are taken altogether (Figure 1, Table 1) and when considering only obese children (p < 0.05). Average BMI was substantially unchanged after TEP, while it significantly increased after DT (p < 0.05).

Variations in obesity degree (overweight, obese, non severely obese and severely obese) are summarized on Table 3. The difference in obesity degree reduction observed between the two groups is statistically significant in favor of children who followed the TEP only for obese subjects (**X**^2 ^test p < 0.01).

**Table 3:** Changes of obesity degree (Overweight, obese, non severely obese, severely obese) in the two groups, from baseline to the end of follow-up

	**Decrease of obesity degree**		**Increase of obesity degree**	
	**TEP**	**DT**	**TEP**	**DT**

Overweight	25%(2/7)	29.17% (7/24)	12.5%(1/7)	33.33%(8/24)
Obese	39.74%(31/78)*	19.75%(16/81)*		
Non severely obese	38.5%(10/26)	19.6%(9/46)	11.5%(3/26)	15.2%(7/46)
Severely obese	40.4%(21/52)	20%(7/35)		

*p < 0.01

TEP : Therapeutic Education Program

DT: Dietetic Therapy

**Figure 2:**
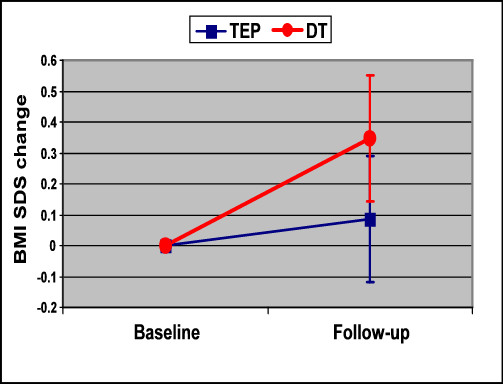
**BMI SDS changes from baseline to follow-up in Negative children, following TEP and DT**. TEP: Therapeutic Education Program. DT: Dietetic Therapy. Negative: children with a BMI% increase ≥ 10% from baseline at follow-up. Negative after TEP: number children 10. Negative after DT: number children 27. BMI SDS: BMI Standard Deviation Score.

At follow-up, the BMI % and BMI SDS of negative children showed a lower increase in the study group compared to the DT group, but the difference was not statistically significant (Figure 2).

On the other hand no significant difference was found between males and females, notwithstanding the males had a higher overweight degree both at baseline and after follow-up. Even if among children with negative results, males comprise only 40% in both groups, the gender difference does not appear to be significant.

Usually families refuse to enter the program because they do not like to take part in training groups. Despite the low compliance for the treatment and the very high drop-out percentage in pediatric age [4,20], periodical telephone calls greatly reduced the drop-out percentage in TEP group (2 out of 85 children); 22% of children belonging to the TEP group received only one telephone follow-up. The drop-out percentage in the DT group, that did not receive any telephone follow up, was 45.7%.

We did not observe any difference between the anthropometrical measures (height and weight) reported by families and those made by the pediatrician during the clinical consultation following the phone call (25%).

Initial questionnaires investigating parents' opinion and feelings about the TEP show a good level of acceptance and a high level of satisfaction. Final questionnaires, carried out only in families with a 3-year follow-up, show an improvement in life style of children without a worsening in their quality of life (Table 4).

**Table 4:** Evaluation Questionnaires

**Time**	**Assessed issue**	**Good**	**Bad**
**Therapeutic Education Session** n. 85	• Appropriateness	n.84 (99%)	n.1
	• Completeness	n.84 (99%)	n.1
			

**1**^st ^**year Clinical Assessment** n. 56	• Effectiveness of therapeutic		
	Education and Self-help book	n.52 (93%)	n.4
	• Satisfaction	n.52 (93%)	n.4

**3**^rd^**year Clinical Assessment** n. 40	**Improvement in**		
	• Eating behavior	n.39 (98%)	n.1
	• Physical activity pattern	n.35 (88%)	n.5
	• Life Quality	n.39 (98%)	n.1

Results of questionnaires assessing treatment satisfaction, life-style and life-quality, as perceived by children and their parents, in different moments of the follow up.

## Discussion

The stability of clinical outcomes observed after a 3-year follow-up seems to confirm the efficacy of "*The Balloon Game*".

In fact, if we accept that the weight reduction or stabilization is an acceptable therapeutic result, both the TEP group, with the 85%, and the DT group, with the 66%, achieved the goal. However, the TEP group hit the target without any dietary restraint and its negative consequences [33]. Moreover, "*The Balloon Game*" did not seem to cause any adverse effect, such as growth delay, additional psychological troubles or eating disorders.

Our TEP was conceived to contend with three main problems: the growing number of obese children seeking advice in our Department, the lack of human resources and the need to keep costs under control in public healthcare facilities and the extremely low parent awareness and compliance with therapy [13,34,35].

Furthermore, obese children frequently experience some psychological discomfort, such as poor body image, low self-esteem, weight-related teasing and binge-eating disorders [36-39]. Early-onset overweight is an important risk factor that requires a very attentive and skilful person-centered approach.

We developed an "intensive" and reasonably simple, one-man (i.e., carried out by a single skilled pediatrician), therapeutic strategy for selected obese-overweight children, in the attempt to devise a possible alternative to multi-professional teamwork, that sometimes is not available or may be unpleasant for the children's families.

This program could produce good results in overweight and also severely obese children and adolescents without clinically evident personal or familial psychological disturbances. Children who had suspected or clinically evident psychological troubles at the initial assessment (10% of the original group) were excluded from the study.

Five female children (5.6%) who showed "body-image dissatisfaction" or "emotional eating" at initial assessment, received bimonthly behavioral therapy. The occurrence of body-image dissatisfaction or emotional eating disorders was assessed by the pediatrician based on her experience, with the help of a simple clinical interview. However, one of the limitations to this study is that the assessment took place without the use of a standardized clinical interview and/or validated questionnaires.

Observed overweight reduction was good in *obese *and also in severely obese children, but smaller among *overweight *children; perhaps because our TEP did not stress the attainment of an ideal-weight, but the simple adoption of a healthier behavior and life-style. Adolescent children, who usually are at higher risk of drop-out and of treatment failure [3], showed a larger overweight reduction than pre-pubertal children; they also had an improved lifestyle at follow-up.

We do not think that our TEP represents the gold standard for children with severe obesity, who certainly could derive more benefit from other therapeutic approaches. However, when other choices are neither available nor accepted, our TEP seems to provide acceptable results also in these cases.

A higher, yet not statistically significant prevalence of negative results was observed among younger children and females. A rapid weight gain pattern during early childhood ("adiposity rebound") and during sexual development in females are risk factors for obesity for many reasons (genetic predisposition, life-long changes in the appetite regulating centers, etc.) [40]. Therefore, early therapeutic interventions aimed at controlling weight gain in early childhood, even if often refused, should be carefully considered in order to assure lasting results [9].

After TEP, negative children were significantly fewer than those undergoing DT (11.8 vs. 25.7%) (p < 0.05). The same occurred with severely obese children, decreasing by 19% after TEP and remaining unchanged after DT (p < 0.01) (Table 2), with a consequently lower expected risk of metabolic syndrome and related cardiovascular outcomes [41]. After TEP sessions, the drop-out rate was reduced by periodic phone calls.

Our results seem similar to those obtained in more intensive trials, usually involving only selected and strongly motivated families [6,7].

Moreover, it should be underlined that, compared to DT, TPE does not involve any prescription, but simply trusts the children and their families, promotes their self esteem and puts them in the condition of making free and responsible choices. By contrast, DT adopts a dietician-centered, prescriptive approach, that tends to substitute parental role and to reduce parents' self esteem, rather then helping them to develop a self-determined and responsible attitude toward their child's health.

During the study period no information had been exchanged between the two therapists.

A major problem was that only 31% of all obese children's families coming to our Department, mostly the more severe, agreed to adhere to the TEP. In order to increase their motivation and participation we are now planning to involve family pediatricians of our district, so that they promote the TEP and its goals among obese children's families. A recent study by Quattrin [9] shows that families and pediatricians usually refer children to nutritional medical facilities for being underweight rather than overweight; indeed, in Italian rural mentality, "well nourished" children are still considered "healthy" children. However, it is also possible that some pediatricians might consider current therapeutic approaches to obesity worthless.

Our approach, based on the commitment and autonomy of children and their caregiver [42], led to high rates of satisfaction and better treatment results. The first aim of our program is helping children to find a healthier lifestyle, thereby preserving the quality of life. Indeed, therapeutic education is "designed to enable a children (and families) to manage the treatment of their condition and prevent avoidable complications, while maintaining or improving quality of life" (WHO, 1998) [43].

## Conclusion

"*The Balloon Game*" is a retrospective, non-randomized nor case-controlled clinical study. Therefore, our data cannot provide definitive evidence that our three-session, non-dietetic, therapeutic education program is effective in the treatment of children obesity. Indeed, only randomized and case-controlled clinical studies will help to elucidate its real efficacy and reproducibility in different healthcare settings. Moreover, it would be important to better understand the influence of family social and economical conditions and the degree of obesity (initial BMI%) on the final results of our study.

As it stands, "*The Balloon Game*" could offer a possible therapeutic alternative at least for those children with overweight or non-severe obesity, who enjoy a medium-high socio-economic condition and are without psychological troubles. Additionally, it could be employed in the field of prevention and primary care.

Nevertheless, we think that it could offer some help also to severely obese children, who usually find major problems in attending long and difficult therapeutic programs. Indeed, a more "friendly" and "sustainable" therapeutic proposal might help them to recover enough self-confidence and trust in healthcare professionals, enabling them to participate in a longer and more rigorous therapeutic program.

In conclusion, despite some evident limits, we think that our experience might suggest that:

• A family-based Therapeutic Education approach to obesity, making children and their families able to cope with their problem in an conscious and independent way, may produce results that last longer than those obtained with standard dietetic treatments; in addition, it can reinforce their motivation and self-efficacy, improving their quality of life and promoting their empowerment.

• A "sustainable" therapeutic education program (i.e., requiring limited time and professional resources), carried out by a single, skilled pediatrician, might represent a feasible and convenient therapeutic solution for selected obese children when Behavioral Therapy is not available or teamwork is poor or not possible.

• Healthcare professionals dealing with pediatric obesity should take into account new personalized standards in the evaluation of therapeutic outcomes, carefully looking both at parents' weight and the child's weight variations before treatment, and also paying attention to the negative consequences of dietary restraint.

• The drop-out rate of obese children/adolescents can to be reduced by periodic telephone calls.

## List of abbreviations used

TEP: Therapeutic Education Program

DT: Dietetic Therapy

BMI: Body Mass Index

BMI-SDS: BMI Standard Deviation Score

## Competing interests

The author(s) declare that they have no competing interests.

## Authors' contributions

RT contributed to the conception, design and conduct of the study and to the interpretation of the data, as investigator and corresponding author, she had full access to all the data in the study and takes responsibility for the integrity of the data and the accuracy of the data analysis. RM contributed to the conception of the study, critically interpreting the meaning of its results. SP contributed to data collection, organization, analysis and their interpretation. GG provided to design of the study and advice on the statistical analysis of the data.

Each author has been involved in the conception and design of the study, or the analysis of the data and has participated in the writing of the manuscript. All authors read and approved the final manuscript.

## Pre-publication history

The pre-publication history for this paper can be accessed here:


